# Exosomes—the enigmatic regulators of bone homeostasis

**DOI:** 10.1038/s41413-018-0039-2

**Published:** 2018-12-07

**Authors:** Minhao Gao, Weiyang Gao, J. M. Papadimitriou, Changqing Zhang, Junjie Gao, Minghao Zheng

**Affiliations:** 1 Centre for Orthopaedic Research, Faculty of Health and Medical Sciences, The University of Western Australia, Nedlands, WA 6009 Australia; 2 Perron Institute for Neurological and Translational Science, Nedlands, WA 6009 Australia; 3 Department of Orthopaedics, The Second Affiliated Hospital and Yuying Children’s Hospital of Wenzhou Medical University, Wenzhou, China; 4 Pathwest laboratory, Perth, WA Australia; 5 Department of Orthopaedics, Shanghai Sixth People’s Hospital, Shanghai Jiaotong University, Shanghai, China

## Abstract

Exosomes are a heterogeneous group of cell-derived membranous structures, which mediate crosstalk interaction between cells. Recent studies have revealed a close relationship between exosomes and bone homeostasis. It is suggested that bone cells can spontaneously secret exosomes containing proteins, lipids and nucleic acids, which then to regulate osteoclastogenesis and osteogenesis. However, the network of regulatory activities of exosomes in bone homeostasis as well as their therapeutic potential in bone injury remain largely unknown. This review will detail and discuss the characteristics of exosomes, the regulatory activities of exosomes in bone homeostasis as well as the clinical potential of exosomes in bone injury.

## Development: Exosomes guide bone development and offer clinical promise

Vesicles known as exosomes may prove to be valuable clinical tools once their function is clarified. Exosomes were discovered in the 1980s but not observed in bone tissue until 2003. Minghao Zheng of the University of Western Australia, together with colleagues elsewhere, has reviewed the biology of exosomes, their role in maintaining bones, and their potential clinical uses. Exosomes carry lipids, proteins, and nucleic acids between cells. They are released by every type of bone cell, with the role of each exosome determined by its specific contents. Exosome-mediated crosstalk is involved in regulating bone remodeling, and exosomes have also been implicated in myelomas. Recent work has shown that exosome treatment can improve fracture healing. The authors conclude that a better understanding of the role of exosomes in bone homeostasis will unlock their significant clinical potential.

## Introduction

Bone is a composite tissue, whose matrix consists of proteins and minerals, and which constantly undergoes modelling and remodeling through the coordination of osteoclasts, osteoblasts, and osteocytes. Osteoclasts, derived from mononuclear hematopoietic myeloid lineage cells, are responsible for bone resorption.^1^ Osteoblasts, accounting for the (4–6)% of total resident cells in the bone, are responsible for bone formation.^2^ Osteocytes, the most abundant cells in bone, are terminally differentiated from the osteoblasts, and are embedded in mineralized bone matrix. Osteocytes play a critical role in sensing mechanical loading and regulate functions of osteoclasts and osteoblasts.^3^

The interaction and coordination of these bone cells are important for maintaining bone homeostasis. Bone formation usually begins with the death of osteocytes.^3^ The apoptotic osteocytes release bioactive molecules, which induce other viable osteocytes to secret receptor activator of nuclear factor κB ligand (RANKL) which is important for osteoclast differentiation.^4^ Subsequently, osteoclast precursors are recruited by chemokines such as monocyte chemoattractant protein (MCP)-1, -2, and -3.^5^ The binding of receptor activator of nuclear factor κB (RANK)-RANKL on the surface of monocytes then initiates osteoclastogenesis.^6,7^ Meanwhile, osteoblasts produce bioactive molecules including macrophage colony-stimulating factor (M-CSF), MCP-1, and RANKL for the further recruitment and differentiation of osteoclast precursors.^5,8^ While resorbing damaged bone, osteoclasts spontaneously secret “coupling factors”, such as insulin-like growth factor (IGF) I and II and transforming growth factor (TGF)-β, which mediate the refill of resorbed lacunae by osteoblast.^9^ Finally, bone formation is completed when the newly mineralized-extracellular bone matrix completely replaces the resorbed bone matrix.^10^

Bone-derived exosomes are considered to be essential for intercellular communication between bone cells. Exosome-mediated transfer of nucleic acid or protein cargos between bone cells can bypass the space barriers between different cells, and plays a vital role in the crosstalk between bone cells regulating bone homeostasis. As the role of exosome is a new mechanism of bone formation and homeostasis, which has only recently emerged, we summarize the characteristics of exosomes, itemise the known functions of exosomes in bone homeostasis, and discuss their potential for clinical applications.

## History of the exosome

### A general history of the vesicular nature of exosomes

Exosomes,^11^ microvesicles,^12^ and secretory autophagosomes^13^ are three typical extracellular vehicles (EVs) identified recently. However, in early studies, there was no detailed classification or understanding of these extracellular vesicles.

Cellular vesicular components were recognised 140 years ago. Under dark-ground illumination, serum-derived particles were first seen by Edmunds in 1877.^14^ The main mass of these particles was then proved as fat in 1939.^14^ Since the function of these particles was unclear, they were just seen as blood dust.^14^ Clearer structure of cellular vesicles was then seen in microscope in 1962.^15^ However, the function of cellular vesicular components remains mysterious until 1969, when the finding of crystals of appetite suggested the participation of cartilage-derived matrix vesicles in calcification.^16^ Five years later, microvesicles in fetal calf serum were detected, which was the last class of EVs detected before exosome was defined.^17^

In 1981, the term exosome was first used for extracellular vesicles ranging from 50 to 1 000 nm.^18^ In 1983, the Stahl group and the Johnstone group reported that exosomes derived from reticulocytes could fuse with the plasma membrane and release their contents through exocytosis.^19^ Then in 1985, the same group provided the electron microscopic evidence for externalization of exosomes.^20^ In 1987, the formation of exosomes was described, and was the first time that the intraluminal vesicles of multivesicular endosomes (MVEs) were mentioned.^21^ The analysis of exosomal characteristic developed quickly in first decade after the exosome was defined. However, the function of exosomes remained largely unknown.

A breakthrough in exosomal investigation took place in 1996 when peptide-major histocompatibility complex (MHC) class II complexes-enriched exosomes released from B cells targeting T cells were detected. This finding first described the role of exosome in cell-to-cell communication.^22^ Following that, dendritic cell (DC)-derived exosomes^23^ and tumor-derived exosomes^24^ were investigated one after the other. These two studies showed the interactions and crosstalk between DCs and tumor cells. DC-derived exosomes could suppress the growth of tumors, and tumor cell-derived exosomes which contained tumor-rejection antigens could be carried by DCs for cross-protection from tumors.^23,24^ These findings were appealing to tumor investigators, and resulted in the generation of numerous reports associated with the tumor-derived exosomes.

**Fig. 1:**
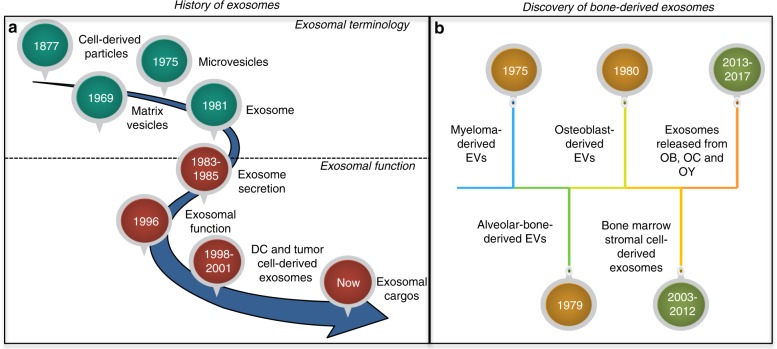
History of exosome research and discovery of bone-derived exosomes. **a** History of exosome research starts from 1877 when serum-derived particles were first detected. **b** Exosomes in bone are derived from osteoclast (OC), osteoblast (OB), osteocyte (OY) and bone marrow stromal cells

The past decade has witnessed an acceleration of exosomal investigations, especially in studies of exosomal function. It is believed that exosomes are the most clearly defined group of secreted membrane vesicles, characteristically containing nucleic acid and proteins for cell signalling.^25^ Physiologically, they are critical to the immune system as they are involved with both stimulatory and tolerogenic responses.^26^ Also, it has been postulated that exosomes could be involved in regeneration, reducing tissue injury and improving tissue repair.^27^ Moreover, they may also be involved in tumor progression^28^ and delivery of inflammatory mediators.^29^ Consequently, the investigation of exosomes is becoming increasingly attractive as they are now suggested to be the key regulators of various cellular and physiological functions (Fig. 1).

### History of bone-derived exosome

**Fig. 2:**
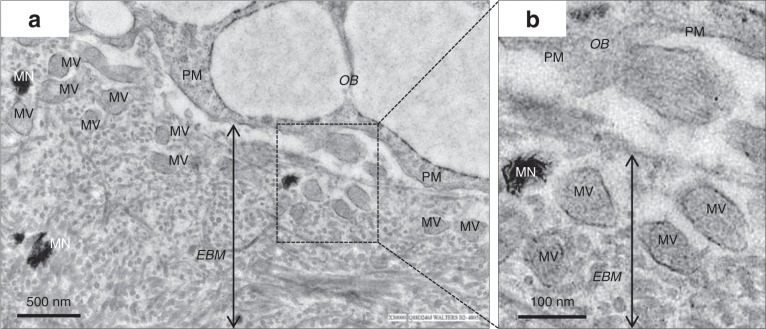
**a** Transmission Electron Microscope (TEM) image of osteoblast (OB) sited in bone matrix. The image shows that abundant of matrix vesicles (MV) are released into extracellular bone matrix (EBM). **b** Insert image demonstrates the diameter of MV from 50 to 150 nm. MN, mineralized nodules; MVB, Multivesicular bodies, late endosomes containing multiple intraluminal vesicles; PM, Plasma membrane

The history of bone-derived exosomes, however, is relatively recent. In 1975, extracellular membrane particles were first found in bone marrows which suggests a possible link between multiple myeloma-derived extracellular vesicles and bone tissue damage.^30^ Then in 1979, normal bone-derived EVs were first mentioned when alveolar bone-derived extracellular matrix vesicles were detected by microscopy.^31^ In 1980, osteoblast-derived matrix vesicles were investigated through ultrastructural techniques. While comparing scanning electron microscopy (SEM) with transmission electron microscopy (TEM), researchers theorised that osteoblast-derived vesicles probably serve as the initial locus of calcification^32^ (Fig. 2).

The first mention of bone-derived exosomes was 20 years after the naming of the exosome.^33^ At the beginning, bone marrow stromal cell-derived exosomes were the focus of bone-derived exosomes. However other bone cell-secreted exosomes were barely mentioned until 2013 when osteoclast precursors were reported to release exosomes.^34^ This initiated the investigation of exosomes from other bone cells. In 2015, the proteome of osteoblast-derived exosomes was for the first time investigated.^35^ In 2016, the characteristics and regulatory activities of osteoclast-derive exosomes were demonstrated.^36^ Then in 2017, osteocyte-derived exosomes and their miRNA contents were demonstrated.^37^ Now, the emerging data of bone-derived exosomes has established the details of exosome-based cell-to-cell interaction in bone.

## Compositions of exosomes

**Fig. 3:**
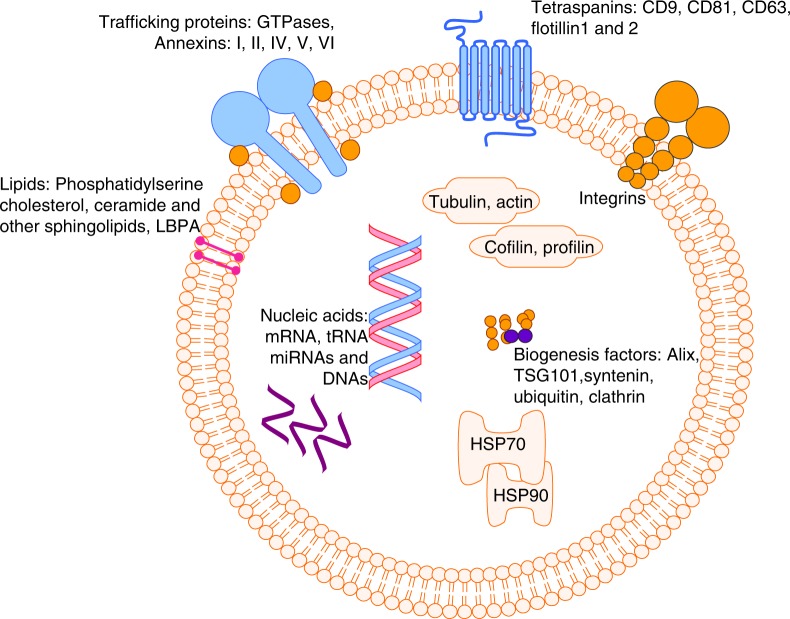
The composition of exosomes. Three main components of exosomes are lipids, proteins, and nucleic acids. Lipids are the main components of exosomal skeleton. They are also involved in exosome biogenesis. Among the lipids, sterols, sphingolipids, and phosiphlipids are dominant ones responsible for cargo sorting. Besides lipids, proteins are also enriched on exosomal surface. Tetraspanins, integrins, GTPases, and annexins are considered to be involved in membrane interaction and trafficking after recognition of surface proteins on recipients (Table 1). There are also various nucleic acid cargos including DNAs, RNAs, and miRNAs detected in the exosomes which determine the specificity of exosomal functions

The function and biological characteristic of exosomes are determined by their specific contents. Among the exosomal components, lipids, proteins, and nucleic acids are three main cargos which determine the specificity of exosomes^38^ and distinguish them from other extracellular vesicles (Fig. 3). A great variety of exosomal cargos have already been identified in exosomes and put together into a database named Exocarta,^39^ which was subsequently integrated into a broader database, Vesiclepedia.^40^ Some examples of exosomal cargos are summarized in (Table 1).

**Table 1:** Molecular compounds in exosomes

Composition of exosomes	Classification	Examples	Functions	References
Proteins	Surface proteins and tetraspanins	CD9, CD63, CD81, CD82, Alix, TSG101	Organizing membranes into tetraspanin-enriched domains (TEMs)and contributing to exosome binding to target cells	^149– 151^
	ESCRT-related proteins	Alix, TSG101	Controllers of exosome secretion via regulating ESCRT machinery	^77, 152^
	Heat-shock proteins	Hsp70, Hsp90	Exosome formation or externalization during the maturation.	^153^
	Rab GTPases proteins	Rab27a, Rab27b Rab35	Involving in MVBs interaction with the plasma membrane	^94, 154^
	Annexins	Annexins I, II, IV, V, and VII	Membrane transport/trafficking	^155^
	Phospholipase	Phospholipase D	Regulating exosome secretion via hydrolyzation of phosphatidylcholine	^156^
	Cytosolic proteins	β-catenin and Elongation factor-1α	Signal transduction and protein translation	^155^
Lipids	Glycerophospholipids	Phosphatidylserine	The activator of negative charge and the recruiter of signalling proteins	^41, 44, 45^
		Phosphatidylglycerol	Involved in transbilayer transport mechanism	^86, 157^
	Sphingolipids	Sphingomyelin	Involved in exosomal membrane construction and cargo sorting	^41, 43^
	Sterol lipids	Oxysterol	Involved in membrane contact between intracellular secretory vesicles and the plasma membrane	^41, 158^
	Neutral lipids	Ceramide	Triggering an exosome biogenesis pathway independent of the ESCRT machinery	^50^
		Free cholesterol	Regulating the biogenesis and cellular trafficking in endosomes	^159, 160^
	Polyglycerophospholipid	BisMonoacylglyceroPhosphate (BMP)	Involved in MVB formation and ILV biogenesis	^161, 162^
Nucleic acids	mRNA	CD2AP mRNA (Kidney disease)	Being biomarkers related to podocyte damage	^61^
		GSTπ1, MGMT, APNG, ERCC1, ERCC2, MVP, ABCC3, CASP8 and IGFBP2 (Tumor)	Involved in drug resistance of tumors	^62^
	miRNA	miR-223 (Tumor)	Promoting cancer invasion	^163^
		miR-146a (Cardiovascular disease)	Mediating regenerative function of cardiosphere	^164^
		miR-155 (Asthma)	Relating to the development of inflammatory infiltration into the lung and to airway remodelling	^165^
	DNA	Double-stranded DNA (Tumors)	Identifying mutations present in parental tumor cells	^58^
		Double-stranded DNA (Tumor)	Carrying mutations identical from partental cells	^70^

The protein compounds selected are mainly responsible for the physiological process of exosomes including exosome formation, interaction and trafficking, whereas lipid compounds are majorly involved in the establishment of exosomal skeleton. Other bioactive compounds of exosomes are short chain nucleic acids including mRNAs, miRNAs and DNAs. They are the main single molecules that regulate recipient cells

### Lipids

Exosomal structure and cargo sorting are largely dependent on lipid composition. Various lipids in exosomes have been investigated in the past decades. In a study of cancer cell-derived exosomes, more than 520 lipids from 36 different classes were identified.^41^ Lipids generally are enriched in exosomal membranes. The major non-polar lipids in plasma membrane are sterols, which are highly enriched in multivesicular bodies (MVBs) from late endosomes.^42^ Sphingolipids are also important for exosomal membrane construction, in which sphingomyelin is the dominant component, and is also involved in cargo sorting.^43^ Among exosomal membrane phospholipids, phosphatidylserine is of importance for being the activator of negative charge and the recruiter of signalling proteins.^44,45^

Besides contributing to the composition of the exosomal bilayer membranes, lipids in exosomes also play important roles in exosomal trafficking. During the formation of exosomes, the enrichment of sphingomyelin is found in membrane lipid rafts.^46^ As a result of increased sphingomyelin, down-regulation of ceramide and diacylglycerol occurs and finally reaches a balanced proportion in exosomes.^47^ Moreover, lipids play multiple roles in the sorting of nucleic acids and protein. In miRNA sorting, neutral sphingomyelinase 2 is the first molecule suggested to be associated with this mechanism.^48^ Other lipids such as sphingomyelin, ceramide, and sphingosine 1-phosphate have been proved to play important roles in protein sorting mechanisms.^49^ On the other hand, endosomal sorting complex required for transport (ESCRT)-independent exosome secretion is largely dependent on lipids, which are reported to significantly participate in the release of proteolipid-positive exosomes^50^ and Aβ-peptide-bearing exosomes.^51^ Although lipids are not the main participators of exosomal intercellular communication, their roles for maintaining the biological characteristics of exosomes are of importance.

### Proteins

Through proteomic analysis, many proteins have been found in all mammalian exosomes, such as cytoskeletal components (tubulin, actin, cofilin, profilin), annexins (annexins I, II, IV, V, and VII), and the small GTPase family members rab7 and rab11. Among all these exosomal proteins, cytosolic exosomal enriched proteins such as Alix and TSG101, tetraspanins like CD9 and CD63 are the markers for distinguishing exosomes from other extracellular particles (Table 1). The detection of the proteins listed above allows researchers to quickly assess the characteristic of exosomes.^52^ Recent studies have also suggested that the heat-shock proteins (Hsp) are highly prevalent in exosomes. Among them, Hsp40 can improve the protein-folding environment in recipients, and Hsp70 is the up-regulator of pro-inflammatory cytokines.^53^ Protein composition is also crucially involved in ESCRT-dependent cargo sorting during the formation of exosomes. In ESCRT (-0, -I, -II, -III), recombinant human vacuolar protein sorting proteins (VPS proteins) play a major role functioning as membrane binders and cargo recognisers.^54^

Besides the various proteins mentioned above, there are also several other proteins in exosomes that reflect the specificity of cell origin and distinct exosomal functions. For example, latent membrane protein 1 (LAMP1) is highly expressed in exosomes released from nasopharyngeal cancer (NPC)-derived malignant epithelial cells.^55^ Similarly, a specific cell surface proteoglycan, glypican-1 (GPC1), was detected in exosomes from pancreatic cancer.^56^ Collectively, to maintain the specificity of exosomes derived from different donors, various protein cargos must be sorted into exosomes before their release.

### Nucleic acids

Nucleic acids are also enriched in exosomes. Coding RNAs, non-coding RNAs, single-stranded or double-stranded DNAs are all found in exosomes.^57–59^

It is reported that more than 1 600 mRNAs and 700 miRNAs are detected in mammalian cell-derived exosomes. mRNAs contained in exosomes are usually related to cytogenesis, protein synthesis, and RNA posttranscriptional modification.^57^ Exosomal mRNA have been used as biomarkers since they are specific cargos.^60^ In patients with kidney diseases, downregulation of exosomal CD2AP mRNA has been detected in Urine, which can be used for early diagnosis.^61^ Exosomal mRNAs are also suggested to be involved in drug resistance of tumors. Therefore, the detection of exosomal mRNA level may be used to predict optimal treatment options as well as prognosis.^62^ Another recent report suggests that synthetic exosomal mRNA triggers exogenous protein expression, this may be a novel approach for treatment of genetic protein deficiency-related diseases.^63^

Exosomes also contain abundant miRNAs. In the immune system, miRNA-enriched exosomes are released from T-lymphocyte cells, B-lymphocyte cells and DCs, and the miRNAs are involved in the interaction between T-lymphocytes and antigen-presenting cells.^64,65^ In several tumors, exosomal miRNAs participate in tumor growth,^66^ metastasis, and drug resistance.^67^ Since specific variation of exosomal miRNAs can be detected in some diseases, exosomal profiling can be used as a tool for disease detection.

Exosomal DNA studies began much later than that of RNA, consequently, there is less information available in the literature. To the best of our knowledge, both single-stranded and double-stranded DNAs are contained in exosomes.^58^ Evidence has suggested that carrying cytoplasmic DNAs in exosomes protects against cell senescence and cell death caused by DNA injury. Cells can secret exosomes and remove harmful DNAs to extracellular matrix.^68,69^ However, there is only limited data to elucidate the function of double-stranded DNA in exosomes, and only little is known about the contribution of single-stranded DNAs. In a study of cancer cells, double-stranded DNA is reportedly being used to identify the mutations in cancer cells.^58^ Intriguingly, the expression of DNA cargo in tumor cell-derived exosomes is much higher than that in normal cell-derived exosomes, suggesting that tumor cells can modify target cells via the transfer of DNAs.^70^ There is still long way to go for the complete understanding of the role of exosomal DNA since the mechanism of chromosomal DNA sorting within intralumenal vesicles (ILVs) is still largely unknown.^71^

## Exosomal trafficking

**Fig. 4:**
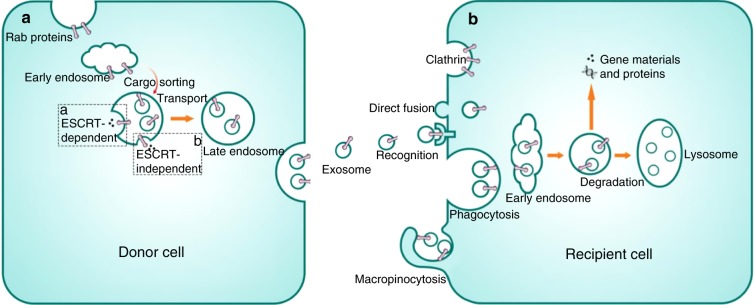
Exosome release and uptake in cells. **a** Exosome formation starts with the formation of early endosome. Subsequently, ESCRT-dependent mechanism (**a**), which consists of four multiprotein subcomplexes (ESCRT 0, I, II, and III), or ESCRT-independent mechanism (**b**), which relies on tetraspanins-associated dynamic membrane platform, mediates the maturation of exosomes. After the fusion of late endosome, which contains mature exosomes, to the cell plasma membrane, exosomes are released into extracellular matrix. **b** Exosome uptake begins with the recognition of specific surface proteins of target cells. Subsequently, they are internalized through several internalization pathways. After that, exosomes can either release their cargos to exert their functions or be directly degraded by lysosome for recycling

Exosomal trafficking involves three distinct mechanisms: cargo sorting, exosome release and exosome uptake (Fig. 4). During the generation of the endosomal machinery, ILVs, the early stage of exosomes, are formed through inward budding. Together with the sorting of specific proteins, lipids and nucleic acids into ILVs, the formation of MVEs results.^72^ Subsequently, MVE fuse with the cell membrane leading to the secretion of exosomes. Following that, the surface binding protein activates the uptake of exosomes in the recipient cells.^73^ Finally, as endocytosis progresses, exosomes release their contents which may influence regulatory processes or they may be degraded in lysosomes.^74^

### Sorting cargos into exosomes

Sorting of protein into exosomes relies on specialized mechanisms, which ensure the specificity of exosomes for various intracellular communication purposes. Here, the ESCRT system, constituted of four multiprotein subcomplexes (ESCRT 0, I, II, and III) appears to be the main mechanism for exosomal formation.^75^ ESCRT 0, I, and II are responsible for recognizing and sequestering ubiquinated membrane proteins at the endosomal membrane, and ESCRT III is responsible for membrane budding and repartition of intraluminal vesicles.^76^ Categorizing of exosomes, however, appears to be a part of cargo ubiquination and only specific ESCRT segments are involved.^77^ The sorting of membrane proteins of the syndecan family into exosomes is regulated by an ESCRT accessory protein Alix through the cytosolic adaptor syntenin.^74^ Alix then binds to ESCRT III which is in control of ILV formation at the MVEs.^78^ Lateral involvement of heparin sulfate polysaccharide chains was reported to determine syndecan complex formation, which are degraded into shorter ones by heparinase activity in endosomes, favoring clustering of syndecans.^79^ Heparinase-induced recruitment is also believed to incite the binding of syndecan cytoplasmic domains to PDZ domains of syntenin, leading the sorting of proteins via Alix-ESCRT pathway.^78,80^

ESCRT-independent protein sorting is another important pathway for exosomal formation. This process requires the formation of a tetraspanins-associated dynamic membrane platform, where cytosolic and transmembrane proteins exert their ability to accept specific proteins into ILVs.^81^ Examples can be seen in CD63-induced endosomal sorting in melanocytes,^82^ and in tetranspanins-dependent recruitment of cholesterol-contained cone-like structures for inward budding.^83^ Although ESCRT-independent protein sorting is different to its counterpart, they both undergo cargo clustering and membrane budding.

Nucleic acid sorting, however, relies on a different mechanism. While DNA sorting is still largely unknown, RNA sorting is concluded previously. Loading RNA into exosomes begins with the formation of the raft-like region.^84^ Subsequently, anionic phospholipids are enriched in the raft-like region of exosomes,which then recruits neutral sphingomyelinase 2 to produce ceramide molecules, an indispensable factor for RNA sorting.^85–87^ Binding of RNAs to the raft-like region is dependent on differential affinity of RNA motifs,^88^ and randomly structured RNAs can bind to rafted domains with a 20-fold higher affinity. Once binding to the budding in the raft-like region, the RNA becomes encapsulated into ILVs and then released into the extracellular space within this vesicle.^84^

### Release of exosomes

The greatest difference in the exocytosis pathway between exosomes and other extracellular vesicles (autophagosomes and microvesicles) is that exosomes are dependent on late endosomes for their release,^74^ and fusion of the MVBs, the late endosomes containing ILVs, with the plasma membrane is the last step before the exosomes are secreted to extracellular matrix. During this phase SNARE proteins and synaptotagmin family members are the main mediators.^89^ Exosomal exocytosis requires SNARE complexes, consisting of syntaxin 7, synaptotagmin 7, and VAMP 7. The SNARE complex is activated by upregulation of intercellular calcium which is Rab protein-dependent.^90^ Subsequently, vesicle (v)-SNAREs and target (t)-SNAREs promote the apposition of budding vesicles and cell membranes.^91^ After the coupling of v-SNAREs and t-SNAREs, the chaperone ATPase N-ethylmaleimide-sensitive factor (NSF) and soluble NSF attachment proteins (SNAPs) catalyze the disassembling of SNARE complexes, leading to the release of exosomes.^91^

Another key factor for exosomal release involves Rab proteins. They are a family of more than 60 proteins which participate in vesicle budding, cytoskeleton interaction and tethering of the receptor compartment to the membrane.^92^ Several examples revealed their participation in exosomal release. In oligodendroglia, Rab 35 was found to participate in PLP (genuine myelin proteins)-bearing exosomal secretion.^93^ Moreover, Rab 27A and Rab27B have been linked to MVBs interaction with the plasma membrane.^94^ These Rab proteins are thought to participate in the eventual fusion of the membranes of exosomes and the plasmalemma of donor cells, resulting in the exocytosis of exosomes.^95^

### Uptake of exosomes

The fusion of exosomes with recipient cells relies on the interaction of vesicular ligands with cellular receptors, such as tetraspanins, integrins, and intercellular adhesion molecules (ICAMs), which induce the binding of exosomes to the surface of target cells. The recognition of surface proteins is the first step during exosomal internalization.^96^ Compelling evidence has proved that exosomal uptake is highly dependent on the signalling status of target cells and of exosomal surface proteins.^11,97,98^

During exosomal internalization, various pathways, including those of endocytosis, phagocytosis, micropinocytosis, and membrane fusion, are shown to participate.^99^ Among them, endocytosis seems to be the commonest way for exosomal uptake. This is a quick process occurring within 15 min.^100^ The most distinctive part of exosomal endocytosis is inward budding of the plasmalemma, which is dependent on the participation of caveolin^101^ and clathrin.^102^ By contrast, during macropinocytosis, exosomes are attached to a highly ruffled region on the cell surface and then taken in via the internalization of the whole region.^103^ This process is similar to phagocytosis.^104^

Moreover, exosomes can also directly fuse their membrane with the plasma membrane of target cells.^105^ This is dependent on two steps of intermediates: hemifusion structures and fusion pores.^106,107^ In most cases hemifusion structures are suggested to be lipid mixture without content mixing which represents the content of outer leaflets but not the inner leaflets of the two bilayers.^106^ Fusion initiates from the formation of fusion stalk, a point-like membrane protrusion of outer leaflet that establishes an hourglass-like connection between the apposed monolayers.^108^ Then an immediate contact of proximal leaflets leads to the formation of hemifusion stalk where leaflets fused and distal leaflets unfused. Finally, a fusion pore opens in the hemifusion diagram dependent on the expansion of stalk,^109^ where a connection between apposed membranes leads to the release of secretions.^107^

## Exosomes in bone homeostasis

Bone homeostasis is of critical importance and relies on the transfer of active molecules between cells, which are summarized in (Table 2). Previous studies have suggested a direct interaction with secretion exchange among bone cells to occur.^110,111^ Recently, however, compelling evidence has emerged to show the regulatory activities that exosomes exert in bone remodelling. Almost all bone cells have been suggested to secret exosomes, and the relationship between bone remodelling and bone-cell derived exosomes is now well documented. Published reports have suggested that transfer of exosomal specific proteins, mRNA and miRNA is the main mechanism for exosome-mediated bone remodelling. This crosstalk establishes a novel network for cell-to-cell interaction during bone homeostasis.^112^

**Table 2:** A summary of function and application of bone-derived exosomes

Features	Target cells	Effect	Bioactive factors involved	Potential application	References
Bone mesenchymal stem cell-derived exosomes	Bone mesenchymal stem cells	Promoting the differentiation of MSC via downregulation of the miR-29b and Notch gene expression	Fas proteins	Improvement of osteopenia	^166^
	Bone marrow-derived mesenchymal stem cells	Enhancing the osteoinductivity of β-TCP through activating the PI3K/Akt signaling pathway of hBMSCs	Tricalcium phosphate	Repair of bone defects	^167^
	Osteoblasts	Promoting osteogenesis via direct transfer of osteogenesis-related genes and proteins	miR-196a, miR-27a and miR-206	Bone regeneration	^115^
Osteoblast-derived exosomes	Bone mesenchymal stem cells	Promoting osteogenic differentiation via up-regulation of β-catenin	miR-667-3p, miR-6769b-5p, miR-7044-5p, miR-7668-3p and miR-874-3p	Bone tissue engineering	^113^
	Osteoclast precursors	Promoting osteoclastogenesis via binding Rank on the osteoclast precursor surface	RANKL	Bone remodelling	^120^
	Osteoclasts	Inducing the apoptosis of osteoclast	RANKL	Detection of osteoclast-poor osteopetrosis	^125^
Osteoclast-derived exosomes	Osteoclasts	Inhibiting osteoclastogenesis via the prevention of RANK-signalling pathway	RANK	Treatment of bone diseases and injury	^121^
	Osteoblasts	Inhibiting osteoblast by targeting activity3’-untranslated region (UTR) of ATF4 mRNA to	miR-214-3p	Improvement of osteopenia	^36^
Osteoclast precursors (Monocyte)-derived exosomes	Mesenchymal stem cells	Inducing differentiation of MSC cells into osteoblast	Not mentioned	Bone regeneration	^34^
Osteocyte-derived exosomes	Osteoblasts	Regulation of osteoblastic differentiation via targeting wnt / β-catenin-signalling pathway	miR-218	Improvement of skeletal muscle loss-related osteoporosis	^126^
	Osteoblasts	Activation of osteogenesis	Sclerostin, RANKL, and osteoprotegerin	Repairation of tibial stress injury	^168^
Bone marrow adipocytes-derived exosomes	Osteoblasts	Inhibition of osteogenesis via the transfer of anti-osteoblastic genes	miR-138, miR30c, miR125a, miR-125b, miR-31	Improvement of osteoporosis	^169^
Bone marrow stromal cells-derived exosomes	Bone mesenchymal stem cells	Inducing stem cell differentiation	Type I collagen and fibronectin	New biomimetic tool for regenerative medicine	^146^

Individual cell specific derived exosomes have distinct roles in bone homeostasis

### Exosome induces osteogenic differentiation of mesenchymal stem cells (MSCs) and osteogenesis

**Fig. 5:**
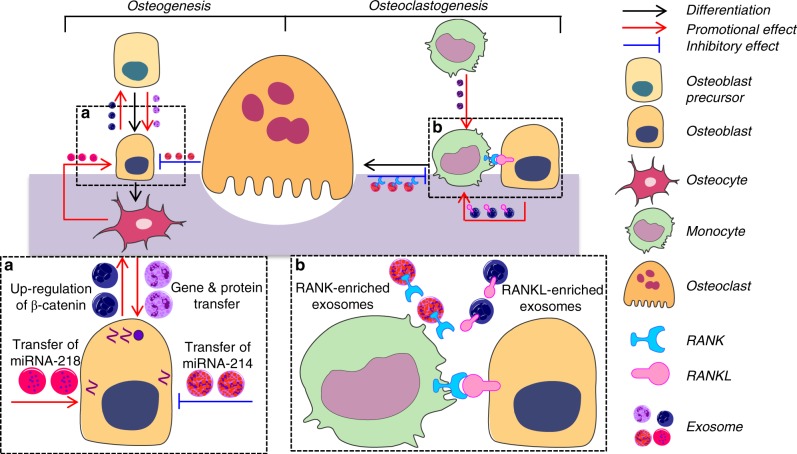
Network of interaction between bone cells via exosomes. **a** Monocyte is the precursor of osteoclast, the differentiation of which relies on RANK-RANKL binding. While monocyte-derived exosomes and RANK-enriched exosomes released from osteoblast can activate osteoclastogenesis, osteoclast secret RANKL-enriched exosomes competitively inhibit this process. **b** Osteogenesis is regulated by several genes which can be transferred via crosstalk. Myostatin-modified osteocyte can secrete miRNA-218 enriched-exosomes to activate osteoblast differentiation. Besides, up-regulation loop can be seen between osteoblast and its precursor via the release of exosomes. However, osteoclast-derived exosomes play an inhibitory role in osteogenesis. Collectively, osteogenesis and osteoclastogenesis can be induced by exosomes derived from various bone cells whereas it seems that only osteoclast-secreted exosomes inhibit these two processes indicating their special role in bone homeostasis

Bone remodelling is a complex process, which is mainly associated with two steps: osteoclastogenesis (for clearance of damaged bone tissues) and osteogenesis (for bone formation). It has been shown that exosomes are crucially involved in these two steps (Fig. 5).

During the process of bone formation, exosomes are suggested to involve in osteogenic differentiation of MSCs. Monocyte-derived exosomes are important stimulators for osteoblast differentiation.^34^ Fusion of these exosomes with MSCs can trigger the up-regulation of two osteogenic markers: RUNX2 and BMP-2.^34^ Intriguingly, newly formed osteoblasts can also secret exosomes to affect their progenitor cells. A group of researchers^113^ found that mature osteoblast-derived exosomes could trigger variation of miRNA expression profiles which, in turn, cooperatively inhibit the expression of Axin1, a central component of Wnt signalling pathway. As a result, β-catenin was up-regulated leading to the enhancement of osteogenic differentiation.

Osteogenesis is also dependent on exosomal functions. Before differentiating into osteoblasts, osteoblast precursors secret exosomes to promote osteogenesis.^114^ During fracture healing, bone marrow stem cell-derived exosomes express MCP-1, MCP-3, SDF-1, angiogenic factors, mRNAs and miRNAs and cooperatively contribute to bone remodelling.^114^ They probably also enhance osteoblast proliferation and differentiation by upregulating osteogenesis-related proteins (RUNX-2, ALP, OCN, and OPN), as well as several genes (miRNA-196a, miRNA-27a, and miRNA-206.^115^ Enhancement of osteoblast proliferation induced by MSC-derived exosomes has also been reported and that the MAPK pathway may be a key factor in exosome-mediated osteoblast activity.^116^

In addition, exosomes derived from osteoblasts and osteoclasts are also involved in osteogenesis. Osteoblasts can secret exosomes to enhance osteogenesis and Let-7-enriched exosomes derived from osteoblasts have been reported to enhance osteogenesis by regulating AT-hook 2 (HMGA2) and AXIN2.^113,117^ By contrast, osteoclast-derived exosomes act as inhibitors of osteogenesis. Exosomal miR-214-3p was suggested to be involved in the inhibition of osteoblast activity by targeting the 3′-untranslated region (UTR) of ATF4 mRNA. The exosomal transfer of miR-214-3p from osteoclasts to osteoblasts was also detected in vitro and thus triggered the reduction of bone mass in mice models.^36,118,119^

### Exosome induces osteoclastogenesis and bone resorption

It is widely accepted that osteoclastogenesis is the basis for bone resorption. The classical osteoclastogenesis model is based on the direct interaction between different bone cells. However, recent studies have suggested a novel mechanism dependent on crosstalk phenomena. Initially, osteoblasts secret RANKL-enriched exosomes which targets monocytes. The RANKL-RANK binding on the monocyte surface then activates osteoclastogenesis.^120^ This process can be augmented by MSC-derived exosomes that can upregulate the expression of Nfatc1, Trap, and Ctsk. While osteoclast differentiation is initiated, the mechanism that controls the number of osteoclasts is initiated. This can be mediated either by osteoclast-derived exosomes or osteoblast-derived exosomes.^121–123^ Newly formed osteoclasts release RANK-enriched exosomes, and these exosomes can either directly fuse to osteoblasts or competitively bind RANKL in the extracellular matrix to regulate the formation of osteoclasts^121^ (Fig. 5b). Additionally, osteoblasts can release exosomes containing miR-503-3p to inhibit osteoclastogenesis by inactivating the RANK-RANKL signalling pathway.^113,122,123^ Alternately, large numbers of monocytes can secret exosomes to promote osteoclast differentiation.^121^ The end result is that osteoclasts are rapidly recruited during this phase, even though osteoclastogenesis-inhibiting exosomes are constantly released.

During bone resorption, the resorbing ability of osteoclasts can be also affected by exosomes. For example, exosomes derived from serum of osteoporotic, osteopenic or aged patients enhance bone resorption.^124^ When bone resorption is close to completion, abundant RANK-enriched exosomes derived from osteoclasts impede osteoclastogenesis. Finally, RANKL-enriched exosomes that are secreted from osteoblasts can inhibit bone resorption via the induction of osteoclast apoptosis.^125^

### Osteocyte-derived exosomes in bone homeostasis

Compared to the investigation of osteoblast and osteoclast-derived exosomes, studies focusing on osteocyte-derived exosomes are few. Available data show that osteocytes also have the ability to release exosomes,^37^ and there appears to be a link between osteocyte-derived exosomes and bone homeostasis.

A group of researchers have shown that myostatin-modified osteocytic exosomes can regulate osteoblastic differentiation via exosomal miRNA-218, by targeting the wnt/β-catenin-signalling pathway.^126^ Wnt/β-catenin signaling is of great importance in bone homeostasis, involving both bone formation and bone resorption, and is widely believed to be orchestrated by the osteocyte.^127,128^ Previous studies have revealed that Sclerostin and DKK1 were the inhibitors of Wnt signalling by binding to the Wnt co-receptors LRP5/6, thereby contributing to bone loss.^127^ Interestingly, induction of exosomes containing miRNA-218 derived from myostatin-modified osteocyte was also inhibited. These exosomes were then found accepted by osteoblast leading to the up-regulation of sclerostin, DDK1, and RANKL.

Another interesting finding is that osteocytes can secret exosomes in response to mechanical loading. Initially, mechanical stimulation triggers immediate contraction of the actin network which results in Ca^2+^ transients. Simultaneously, mechanical stimulation induces the secretion of osteocytic exosomes, shown by immunostaining with the secretory vesicle marker, lysosomal-associated membrane protein 1 (LAMP-1). This process can also be enhanced by the upregulation of intercellular Ca^2+^. Finally, released exosomes which contain sclerostin, RANKL, and osteoprotegerin target osteoblasts to activate osteogenesis.^129^

### Exosomes derived from tumor cells in bone homeostasis

Exosomes can be released from a variety of cell types. The tumor-cell derived exosomes^130^ can affect bone homeostasis. These effects of cancer cells on bone remodelling provides a new perspective for understanding bone diseases in the course of malignancy.

Tumor cells can spontaneously secret exosomes, and fusion of exosomes to bone cells may trigger either inhibition or abnormal enhancement of bone cell function. Exosomes released from multiple myeloma cells have been proved to support the survival of osteoclast precursors via the down-regulation of TRAP mRNA expression induced by inhibition of caspase-3 activity. Further, enhanced differentiation of osteoclast precursor was observed, which explains the increased bone resorption in myeloma patients.^130^ Enhanced osteoblast activity has been observed as well. It can be induced by the transfer of exosomal miRNA-214-3p which facilitates osteoblastic metastases.^131^ As bone is the initial site for tumor metastases,^132^ Exosomes can also participate in the establishment of bone metastasis, leading to tumor‐induced osteolysis.^133^ In the process of metastasis, exosomes play an important role as they are carrier of miRNA-192, a pivotal factor in tumor induced angiogenic activity.^134^ This is likely to influence pathways involved in the generation of proteases, adhesion molecules, and chemokine ligands, contributing to the metastatic spread of the tumors.

### Exosomes-based clinical applications in fracture healing

**Fig. 6:**
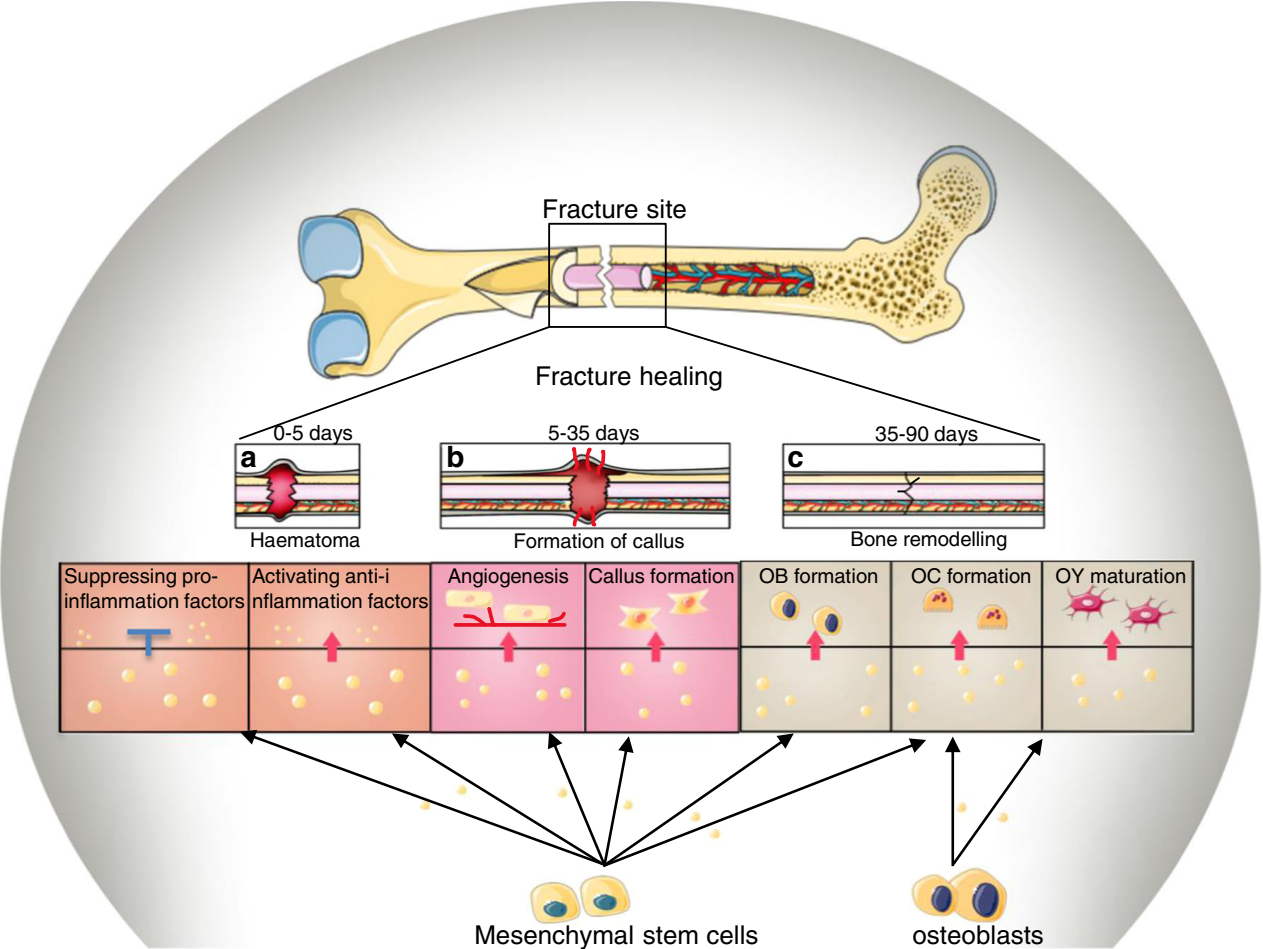
Roles of exosomes in fracture healing at different stages. **a** Mesenchymal stem cell (MSC)-derived exosomes have anti-inflammation property by upregulating anti-inflammation factor TGF-β and suppressing pro-inflammation factors: TNF-α and IL-1β. Thus, exosomes can be used to prevent over reaction of inflammation-induced delay of fracture healing. **b** In the stage of callus formation, exosomes act as promoters by enhancing proliferation and differentiation of endothelial cells as well as the formation of fibroblasts. As a result, enhanced angiogenesis and callus formation induced by exosomes can be detected at this stage. **c** Osteoblast (OB) and MSC-derived exosomes are promoters of bone remodelling. MSC-derived exosomes are suggested to boost the proliferation and liability of OBs. Besides, MSC and OB-derived exosomes are shown to enhance osteoclast (OC) differentiation. OB-derived exosomes are also enhancers of osteocyte (OY) formation by regulating AT-hook 2 (HMGA2) and AXIN2

Recent studies have shown the therapeutic potential of exosomes in different stage of fracture healing, suggesting that individualized strategies can be used to promote bone tissue repair. The initial step of fracture repair is the establishment of new vessels and formation of hematoma at the fracture site where inflammatory cells are being recruited.^135^ Prolonged activation or attenuation of inflammation may lead to excessive bone tissue damage or accumulation of necrotic bone respectively.^136–138^ MSC-derived exosomes are supposed to ideally attenuate inflammation-based delay of fracture healing. By using MSC-derived exosomes, proinflammation factors TNF-α and IL-1β are significantly suppressed, while anti-inflammatory factor TGF-β is increased^139^ (Fig. 6a). Moreover, exosomes are stable carriers for antiinflammation drug delivery. When encapsulated in exosomes, curcumin, an anti-inflammation drug, is more highly concentrated in blood. Moreover, as the drug is more accurately delivered to inflammatory cells due to target specificity of exosomes, there is obvious reduction of unwanted side effects. However, in a certain period of bone healing, inflammation is suggested to be indispensable. Over inactivation of inflammation may lead to delay of fracture healing or even non-union.^140^ Thus, timing of intervention is important. Exosomes also play a role in proinflammatory processes. There is evidence to suggest that acrophage-derived exosomes induce the differentiation of naive monocytes into macrophages.^141^ In this way, recruitment of macrophages, which contain approximately 29 cytokines for tissue repair and inflammation, will relieve an inflammation deficiency-based fracture healing delay.^140^

Tissue repair is the second stage of bone healing when exosomes act as promoters of angiogenesis and bone regeneration (Fig. 6b). MSC-derived exosomes have been reported to contain abundant angiogenesis-related proteins.^142^ The latter enable endothelial cell proliferation and vessel formation.^143^ Interestingly, pro-angiogenesis effect and tissue repair are detected contemporaneously in vitro.^144^ In vivo, MSC-derived exosomes are seen to promote angiogenesis and osteogenesis. Eight weeks after implantation of MSC-derived exosomes, strong formation of vessels and bone tissue is detected in osteoporotic rats compared to untreated controls.^145^ These findings provide a novel approach for enhancing early tissue repair when revascularization and fibroblast proliferation in soft callus occur.^135^ Also, the wide range of exosomal functions may allow the use of MSC-exosomes throughout the whole period of fracture healing.^145^

Bone remodelling at its final stage generally is long-lasting (Fig. 6c). It reaches a degree of homeosteosis between different bone cells. Bone-derived exosomes have been proposed to have a regulatory function on each bone cell type. Osteoclast precursors together with osteoblast-derived exosomes have been detected to promote osteoclastogenesis in vivo,^121,125^ thus, could be used to boost the clearance of damaged tissue. During bone remodelling, MSC-derived exosomes have been shown to promote this process.^146^ In a femur fracture model of CD9^−/−^ mice, which is suppressed in exosome production, there is obvious delay of callus formation leading to retardation of bone union. By local injection of exosomes, however, this retardation is rescued.^114^ Enhancement of cell proliferation and protection from cell death, MSC-derived exosomes could then serve as a powerful tool in bone remodelling.^147^ Such data support the concept that MSC-exosomes-based therapy is ideal for fracture healing for the repair of large bone defects.^148^

## Conclusion

The past decade has witnessed significant progress in the investigation of exosomes as regulators of bone homeostasis, although the function of each of their single molecular species requires further analysis. Whether exosomes are, however, dominant factors in bone homeostasis needs to be further addressed in the future. Such studies will help to better understand the nosogenesis of several exosome-associated bone diseases. Although the introduction of exosomes into clinical practice is not likely to be soon, the perceived power of exosomes in bone homeostasis provides the possibility of novel approaches in the treatment of bone damage and disease.
