# Author Correction: Exosomes—the enigmatic regulators of bone homeostasis

**DOI:** 10.1038/s41413-018-0043-6

**Published:** 2019-01-25

**Authors:** Minhao Gao, Weiyang Gao, J. M. Papadimitriou, Changqing Zhang, Junjie Gao, Minghao Zheng

**Affiliations:** 1 Centre for Orthopaedic Research, Faculty of Health and Medical Sciences, The University of Western Australia, Nedlands, WA 6009 Australia; 2 Perron Institute for Neurological and Translational Science, Nedlands, WA 6009 Australia; 3 Department of Orthopaedics, The Second Affiliated Hospital and Yuying Children’s Hospital of Wenzhou Medical University, Wenzhou, China; 4 Pathwest laboratory, Perth, WA Australia; 5 Department of Orthopaedics, Shanghai Sixth People’s Hospital, Shanghai Jiaotong University, Shanghai, China

**Correction to:** *Bone Research* **6**, Article number: 36; 10.1038/s41413-018-0039-2; published online: 07 December 2018.

In the original publication of this article [1] there is an error in the formatting of Fig. 6. The updated Fig. 6 is published in this correction article. The publisher regrets the error.

**Fig. 6:**
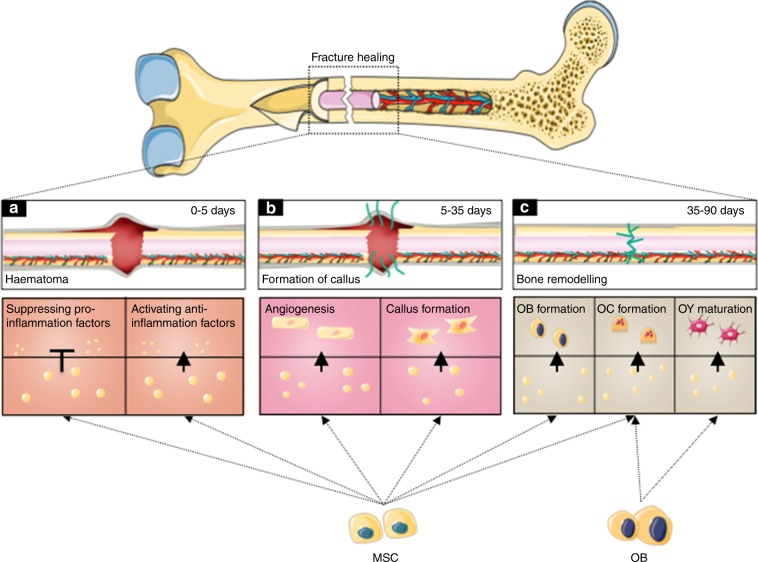
The updated version of Fig. 6 with the correct formatting
